# Use of Sourdough in Low FODMAP Baking

**DOI:** 10.3390/foods7070096

**Published:** 2018-06-22

**Authors:** Jussi Loponen, Michael G. Gänzle

**Affiliations:** 1 Fazer Group, 01230 Vantaa, Finland; jussi.loponen@fazer.com; 2 Department of Agricultural, Food and Nutritional Science, University of Alberta, Edmonton, AB T6G 2P5, Canada

**Keywords:** sourdough, FODMAP, fructan, mannitol, lactobacilli, irritable bowel syndrome (IBS), non-celiac wheat intolerance

## Abstract

A low FODMAP (fermentable oligosaccharides, disaccharides, monosaccharides, and polyols) diet allows most irritable bowel syndrome (IBS) patients to manage their gastrointestinal symptoms by avoiding FODMAP-containing foods, such as onions, pulses, and products made from wheat or rye. The downside of a low FODMAP diet is the reduced intake of dietary fiber. Applying sourdoughs—with specific FODMAP-targeting metabolic properties—to wholegrain bread making can help to remarkably reduce the content of FODMAPs in bread without affecting the content of the slowly fermented and well-tolerated dietary fiber. In this review, we outline the metabolism of FODMAPs in conventional sourdoughs and outline concepts related to fructan and mannitol metabolism that allow development of low FODMAP sourdough bread. We also summarize clinical studies where low FODMAP but high fiber, rye sourdough bread was tested for its effects on gut fermentation and gastrointestinal symptoms with very promising results. The sourdough bread-making process offers a means to develop natural and fiber-rich low FODMAP bakery products for IBS patients and thereby help them to increase their dietary fiber intake.

## 1. Introduction

Fermentable oligosaccharides, disaccharides, monosaccharides, and polyols (FODMAPs) have beneficial and adverse health effects [1]. Oligosaccharides that are not hydrolyzed and absorbed in the small intestine are rapidly fermented by intestinal microbiota in the terminal ileum and the proximal colon [2,3]. Diverse FODMAPs that are fermented by intestinal microbiota consistently cause adverse symptoms when a dose of about 0.3 g/kg body weight, corresponding to about 15 g/day, is exceeded [4,5]. Adverse symptoms include osmotic diarrhea, intestinal distension, and bloating [5,6]. The extent of the adverse symptoms decreases with the degree of polymerization because of the reduced osmotic load of oligosaccharides in the small intestine, and the reduced rate of fermentation [6]. Adverse effects are not described for non-digestible polysaccharides, which are fermented at a much lower rate [7]. Microbiota in the terminal ileum include proteobacteria and lactic acid bacteria as the dominant representatives; ileal microbiota effectively ferment mono- and disaccharides but typically lack extracellular enzymes for hydrolysis of higher oligosaccharides and polysaccharides [6]. The sensitivity of individuals to adverse symptoms caused by FODMAPs is highly variable; adverse symptoms are often linked to irritable bowel syndrome (IBS). The sensitivity to gas pressure and pain varies highly among individuals; moreover, intestinal microbiota adapt toward the fermentation of specific oligosaccharides; this adaptation reduces or eliminates adverse symptoms [8]. Many FODMAPs are conditionally digestible depending on the genetic status of the host. About 35% of humans are lactase-persistent and digest lactose while lactose is a non-digestible FODMAP in the remainder of the population [9]. A substantial proportion of humans are fructose intolerant; the proportion of fructose intolerant individuals among patients with IBS was reported to be over 60% [10,11]. Fructose absorption is highly dependent on the presence of equimolar amounts of glucose as uptake from the small intestine uses the same transport channels [10]. A rare variation in the sucrose-isomaltase gene reduces the digestibility of sucrose, including sucrose in the FODMAPs; this genetic variant also predisposes for IBS [12].

Health beneficial or prebiotic effects of oligosaccharides relate to the bacterial conversion of oligosaccharides to short chain fatty acids [1,13]. These short chain fatty acids increase the energy harvest from carbohydrates that escape small intestinal hydrolysis and absorption, improve intestinal barrier properties and resistance to enteric infections, and exert systemic effects related to inflammation, cognitive functions, and behavior through specific recognition with G-protein coupled receptors (for reviews, see [1,7,13]). Of note, oligomeric fructans, for which health beneficial prebiotic effects were most consistently demonstrated [13], appear also of particular concern for adverse effects in IBS [6]. Adverse and beneficial effects of FODMAPs are thus interconnected and partially related to the same mechanisms, bacterial fermentation. Consequently, a reduction of adverse symptoms in IBS by a low FODMAP diet also increased the luminal pH and reduced the abundance of bifidobacteria and butyrate-producing colonic bacteria [14,15]. While the term FODMAPs indiscriminately includes all oligosaccharides, different compounds were reported to have divergent effects. Supplementation of a low FODMAP diet with β-galacto-oligosaccharides was reported to improve IBS symptoms relative to a low FODMAP diet [16]. In other words, replacement of FODMAPs with different categories of FODMAPs may improve symptoms of IBS without the adverse consequences of a low fiber diet [1].

Wheat and rye are major contributors to the dietary intake of low molecular weight fructans [17] but whole grain products also are major contributors to the intake of dietary fiber [7]. Fermentation processes during baking may allow conversion or degradation of FODMAPs without reducing the overall dietary fiber content of bread [18]. This review aims to summarize current knowledge on the use of conventional and sourdough baking in the production of low FODMAP bread.

## 2. FODMAPs as Contributors to Non-Celiac Wheat Sensitivity?

Non-celiac wheat sensitivity refers to syndromes where components of wheat cause intestinal symptoms. Triggers and mechanisms of the syndrome are poorly described; non-celiac wheat sensitivity is often self-diagnosed or assessed after exclusion of celiac disease and wheat allergy [19,20]. Non-celiac wheat sensitivity overlaps significantly with IBS [20]. Non-celiac wheat sensitivity has also been described as non-gluten wheat sensitivity since gluten apparently is not a major trigger in these symptoms [21]. While a contribution of FODMAPs to symptoms in IBS is increasingly supported by clinical trials, their role in non-celiac wheat sensitivity is not as well documented. FODMAPs and amylase trypsin inhibitors (ATIs) were suggested as likely non-gluten triggers of these symptoms [19,20]. It is likely that *Triticeae* cereals other than wheat, such as rye and barley, are also potential triggers of wheat sensitivity because they also contain fructans and ATIs.

## 3. FODMAPs in Cereals and FODMAP Metabolism in Conventional Sourdoughs

**Figure 1:**
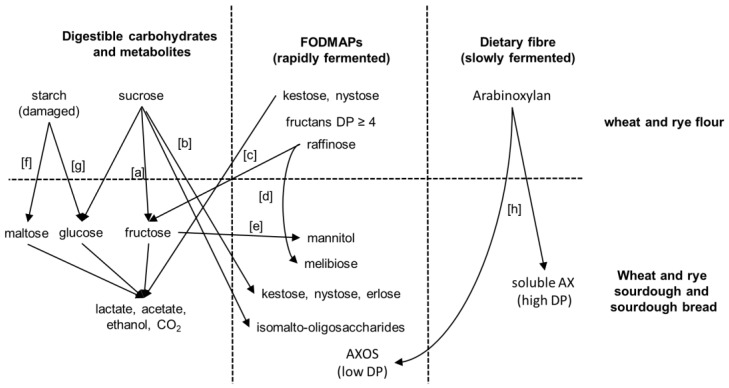
Conversion and generation of fermentable oligosaccharides, disaccharides, monosaccharides, and polyols (FODMAPs) in wheat and rye sourdoughs. Sucrose hydrolysis by yeast invertase or fructosidases of lactic acid bacteria [a]. Oligosaccharide formation by glucansucrases to form isomalto-oligosaccharides, or by fructansucrases to form kestose, nystose, and erlose from sucrose [b]. Kestose and nystose degradation by yeast invertase or by intracellular (phospho)-fructosidases of lactic acid bacteria [c]. Raffinose conversion by yeast invertase and levansucrase from lactic acid bacteria [d]. Fructose conversion by mannitol-dehydrogenase from heterofermentative lactic acid bacteria [e]. Starch conversion to maltose and glucose by flour amylases and gluco-amylase [f,g]. Exogenous xylanases are used in baking to increase the amount of soluble pentosane (arabinoxylan, AX) to improve bread properties, which can produce low DP arabinoxylan oligosaccharides (AXOS) along soluble high-DP arabinoxylan fragments [h].

Resting grains of wheat and rye contain only low levels of monosaccharides; the major oligosaccharides are sucrose, raffinose, and fructans (Table 1). During sourdough fermentation, amylase and glucoamylase activities of wheat and rye flour release maltose and glucose, respectively, from damaged starch [18]. The fructans of cereal grains are graminan-type fructans, which are oligosaccharides built of mixed-linkage fructose units [22]. Fructans in wheat and rye are concentrated in the outer layers of the grain and have an average degree of polymerization (DP) of 5–6; 1-kestose and nystose account for only a minor proportion of the overall fructans (Table 1) [23]. Additional non-starch polysaccharides include arabinoxylans and β-glucans as the major components, polysaccharides composed of mannose, galactose, and galacturonic acid, and trace amounts of pectin (Table 1). In addition to polysaccharides and FODMAPs that are present in the grain, polysaccharides, oligosaccharides, and polyols can be produced by bacterial activity during sourdough fermentation. An overview of the conversion and production of FODMAPs in sourdough fermentation is provided in Figure 1.

During bread making, the fructans undergo partial degradation due to invertase activity present in yeast. The remaining fructan has a lower DP than the native fructan of flour. Low molecular weight fructans may be under-estimated when analyzing fructan in dough; in addition, they are fermented more rapidly than fructans with a higher molecular weight. The fate of fructans is valid for sourdough fermentation, i.e., grain fructans degrade to some extent but in the case of sourdough, the released fructose is also partially converted to mannitol by sourdough lactobacilli. Mannitol is a polyol that is rapidly fermented by gut microbiota. Thus, for accurate FODMAP quantification, mannitol levels in sourdough breads should also be determined. In the following sections, we outline the carbohydrate metabolism in sourdoughs. This is relevant to understand when the focus is in changes of FODMAPs in sourdough bread making.

In straight dough processes, the dough is fermented with baker’s yeast as the sole fermentation organism; the addition of high cell counts of *S. cerevisiae*, 1–2% biomass corresponding to about 10^8^ cfu/g, achieves leavening after a fermentation time of 2 h or less. In sourdough baking, lactic acid bacteria are used as the second group of organisms; moreover, part of the flour is fermented for an extended period of time. The inclusion of lactic acid bacteria extends the metabolic capacity of the fermentation microbiota; the extended fermentation time strongly enhances the contribution of flour enzymes to the conversion and degradation of dough components [18]. Type I sourdoughs are typically fermented between 15 and 30 °C and they have traditionally been used as the sole leavening agent in bread making. To ensure a sufficient metabolic activity and leavening capacity, type I sourdoughs are propagated through one to three fermentation steps prior to mixing the bread dough [27,32]. Fermentation procedures that use sourdough as the sole leavening agent typically result in ~10% of the flour being fermented for >12 h, 20–30% fermented for >6 h, and all of the flour fermented for 2–3 h, i.e., the time required for dough rest and proofing [33,34]. Fermentation organisms in type I sourdoughs generally include *Lactobacillus sanfranciscensis* and *Kazachstania humilis* (syn. *Candida milleri*) and *S. cerevisiae* or *S. exiguus*. Lactobacilli of the *L. brevis*, *L. plantarum*, and *L. reuteri* groups are also represented in type I sourdoughs [32,35]. Industrial bread production generally includes baker’s yeast as the leavening agent; sourdough fermentations in industrial baking (type I or II sourdoughs) aim at dough acidification to improve the baking quality of rye flour, at supporting the leavening capacity of baker’s yeast, and as baking improver [32,33,34]. Fermentation conditions depend on the technological aim of the fermentation and are often specific for a specific production site; typically, 5–20% of the flour is fermented for >12 h while the remainder of the flour is fermented for ~2 h, corresponding to dough rest, shaping, and proofing [33,34]. Type II sourdough fermentation takes place at around 40 °C and the microbiota typically comprise organisms of the *L. delbrueckii* group (e.g., *L. amylovorans* and *L. johnsonii*) and organisms of the *L. reuteri* group (e.g., *L. reuteri*, *L. pontis*, and *L. panis*) [32,36]. Sourdough microbiota are metabolically active if the sourdough is fermented at the bakery but inactivated if the sourdough is stabilized by drying or pasteurization prior to use in baking [34].

Sucrose is metabolized rapidly by invertase activity of *S. cerevisiae*. Yeast invertase is an extracellular or cell wall-bound enzyme and is secreted in excess of the yeast’s capacity to ferment the hydrolysis products [37]. Sucrose metabolism in lactic acid bacteria is mediated by sucrose phosphorylase or sucrose-1-phosphate hydrolase [38]. Sucrose metabolism and the metabolism of other oligosaccharides in homofermentative lactic acid bacteria is repressed by glucose [39]; in contrast, sucrose conversion in heterofermentative lactic acid bacteria is induced by the substrate but not repressed by glucose [40,41]. Fructose is utilized as a carbon source by homofermentative lactic acid bacteria but used as an electron acceptor for the regeneration of reduced cofactors by most heterofermentative lactobacilli [41,42]. Sourdough lactic acid bacteria also harbor extracellular glucansucrases or fructansucrases, which convert sucrose to indigestible poly- and oligosaccharides. These enzymes are frequently present in *Leuconostoc* spp., *Weissella* spp., and species of the *L. reuteri* and *L. delbrueckii* groups but are also present in other lactobacilli including *L. sanfranciscensis* [43,44]. Glucansucrases convert sucrose to polymeric glucans, isomalto-oligosaccharides, and fructose; fructansucrases catalyze the conversion to levan or inulin, fructo-oligosaccharides, and glucose [44]. Sucrose conversion by glucansucrases and fructansucrases accumulated isomalto-oligosaccharides and fructo-oligosaccharides, respectively, in wheat and sorghum sourdoughs; however, accumulation of oligosaccharides to relevant concentrations is observed only when sucrose is added to the sourdough [45,46]. Glucansucrases and the hydrolase activity of fructansucrases generally also release fructose, which is converted to the polyol mannitol by heterofermentative lactic acid bacteria [41,43]. In traditional sourdough fermentations, mannitol accumulates to 10–20 mmol/kg in wheat and 50 mmol/kg in rye, corresponding to 0.2–0.4% and 0.9%, respectively; the mannitol concentration is increased in direct proportion to the sucrose addition to sourdoughs [47]. *Weissella* spp. are exceptional because the majority of strains do not produce mannitol from fructose [45].

Lactic acid bacteria metabolize raffinose by sequential activity of extracellular levansucrase to convert raffinose to melibiose and fructose or fructan, followed by melibiose transport and intracellular hydrolysis by α-galactosidase. An alternative pathway involves raffinose transport and sequential hydrolysis by intracellular α-galactosidase to convert raffinose to sucrose and galactose and sucrose phosphorylase [48]. Metabolism by extracellular levansucrase with intracellular α-galactosidase is faster than the alternate pathway using two intracellular enzymes, presumably because the disaccharide melibiose is transported faster than raffinose [48]. Raffinose metabolism in heterofermentative lactobacilli is not subject to carbon catabolite repression [49] and the relatively high concentrations of raffinose and raffinose level oligosaccharides in pulse flours are rapidly degraded during fermentation [48]. Type I sourdough microbiota and most strains of *S. cerevisiae* are raffinose negative. Nevertheless, levansucrase from *L. sanfranciscensis* and/or yeast invertase converts raffinose to fructose and melibiose [43,50].

The content of fructans is reduced in straight dough processing to 1–1.5% fructans in wheat bread and about 3% in rye bread [51]. Fructans are not degraded in simulated sourdoughs without microbial activity but invertase activity of *S. cerevisiae* and *Kazachstania humilis* results in partial hydrolysis of flour fructans [52,53]. In a straight dough process, the rate of fructan hydrolysis decreases in the order trisaccharides > tetrasaccharides > pentasaccharides and only a small proportion of higher fructans are degraded [54]. Hydrolysis of fructans is mediated by yeast. However, dimerization of the enzyme reduces the activity towards kestose and nystose and sterically prevents access of oligosaccharides with a DP of more than four to the catalytic site [55]. Metabolism of fructans in lactobacilli is mediated by oligosaccharide transport through the ATP-Bbinding-Cassette transporter MsmEFGK or the phosphotransferase (PTS) system PTS1Bca, followed by hydrolysis through intracellular fructosidases or phospho-fructosidases, respectively [38]. Oligosaccharide transport by MsmEFGK and PTS1BCA is limited to fructans with a DP of four or less [56,57]. Metabolic enzymes for fructo-oligosaccharide (FOS) catabolism are frequent in homofermentative lactobacilli where FOS degradation is repressed by glucose [58] but are very infrequently found in heterofermentative lactobacilli [38,43,49]. Intracellular metabolism of FOS by lactobacilli thus does not contribute to the degradation of fructans in wheat or rye sourdoughs.

In summary, conventional dough fermentations, including sourdough fermentations, result in decreased levels of FODMAPs but may generate FODMAPs from the digestible carbohydrates sucrose and fructose (Figure 1). Low FODMAP baking thus necessitates dedicated approaches, particularly involving fructan- and mannitol-degrading organisms.

## 4. Concepts for Low FODMAP Sourdough Baking

Degradation of fructans with a DP of more than four requires extracellular fructanases. Baker’s yeast *S. cerevisiae* does not express extracellular fructanase. However, *Kluyveromyces marxianus* was suggested as an alternative leavening agent with extracellular fructanase activity [53,59]. *K. marxianus* is maltose negative and most strains do not provide sufficient CO_2_ production for dough leavening; the use of *K. marxianus* in low FODMAP baking thus requires co-culture with *S. cerevisiae* [53] or selection of *K. marxianus* strains with sufficient leavening power and addition of amyloglucosidase to provide glucose for *K. marxianus* metabolism [53,59]. Dough fermentation with *K. marxianus* alone or in co-culture with *S. cerevisiae* allowed production of experimental breads with a low fructan content and a volume and sensory properties matching those of experimental breads produced with baker’s yeast [53,59].

**Figure 2:**
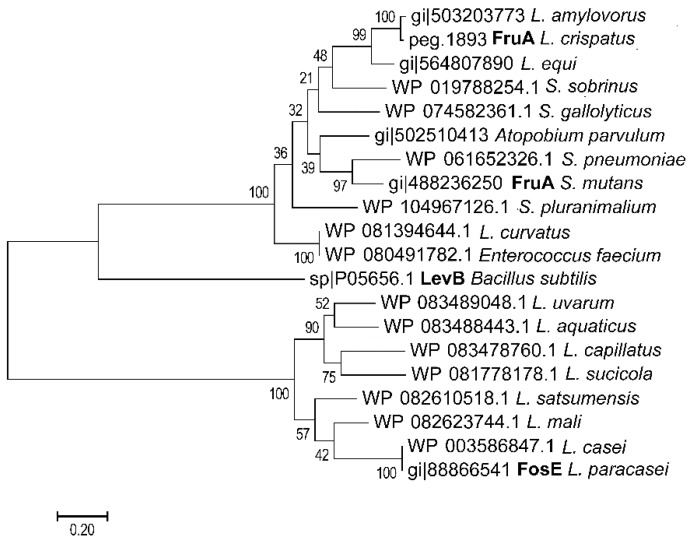
Molecular phylogenetic analysis of extracellular fructanases in lactic acid bacteria by the Maximum Likelihood method. The evolutionary history was inferred by using the Maximum Likelihood method; the tree is drawn to scale with branch lengths measured in the number of substitutions per site. Evolutionary analyses were conducted in MEGA7. Sequences were retrieved by NCBI Blast using the fructanase of *L. crispatus* [62] and the inulinase of *L. paracasei* [60] as query sequence. Sequences from lactic acid bacteria (*Lactobacillales*) with a more than 80% coverage and more than 50% amino acid identity were retrieved and aligned by ClustalW in MEGA 7.0. A levanase of *Bacillus subtilis* was included for comparison. Only one representative sequence for each bacterial species was chosen; sequences of 15 *Streptococcus* spp. which were all similar to sequences of other streptococci were omitted from the tree. The two *Lactobacillus* enzymes that were characterized biochemically are printed in bold.

Extracellular glycosyl hydrolases are exceptional in lactobacilli [38]; accordingly, only a few strains with extracellular fructanase activity have been characterized (Figure 2). The extracellular GH32 β-fructanase FosE was characterized in *L. paracasei* [60]. FosE is an extracellular enzyme that is induced by fructose, sucrose, or inulin but repressed by glucose [60]. BLAST analysis frequently identified homologues of this enzyme in other strains of the *L. casei* group and in few strains of the *L. salivarius* group (Figure 2 and data not shown). The β-fructanase FruA of *Streptococcus mutans* is extracellular with an LPXTG cell wall anchor; the enzyme has less than 40% amino acid identity to FosE ([61] Figure 2). FruA of *S. mutans* plays a critical role in fructan degradation and the virulence of oral streptococci; BLAST analysis frequently identified homologues of FruA in other streptococci (Figure 2). Only five of the more than 1500 genome sequences assigned to the genus *Lactobacillus* harbors FruA homologues; this low frequency suggests that this β-fructanase is not necessary for the lifestyle of lactobacilli but only infrequently acquired by lateral gene transfer. Two of the species with FruA activity, *L. amylovorus* and *L. crispatus*, match species that are typically found in type II sourdoughs.

Type I and type II sourdough microbiota generally include heterofermentative lactobacilli that convert fructose to mannitol. Degradation of mannitol in low FODMAP baking therefore requires mannitol-fermenting lactobacilli. Mannitol metabolism in lactobacilli is mediated by a mannitol-specific PTS system, followed by conversion by mannitol-1-phosphate-dehydrogenase to fructose-1-phospyate [63]. Enzymes for mannitol conversion are present in homofermentative lactobacilli of the *L. delbrueckii*, *L. casei*, *L. plantarum* and *L. salivarius* groups, likely representing trophic relationships with heterofermentative lactobacilli. In analogy to other PTS systems in lactobacilli, mannitol metabolism in homofermentative lactobacilli is repressed by glucose [39,41,58].

**Figure 3:**
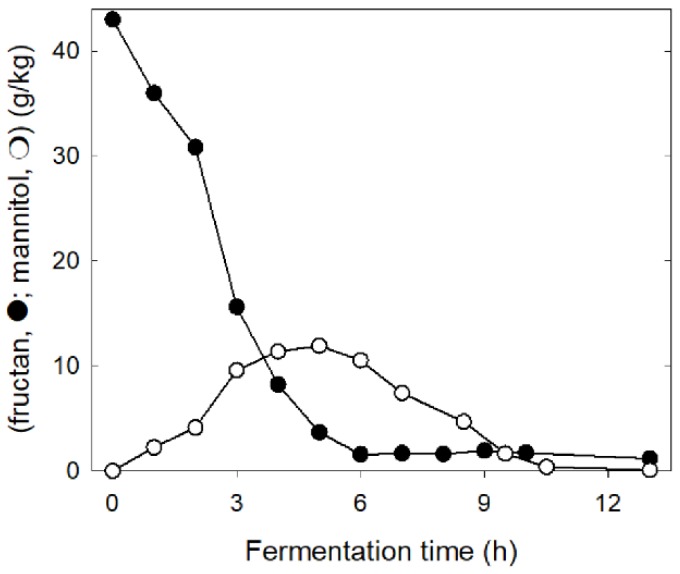
Degradation of fructans (black) and the formation and degradation of mannitol (white) in a type II rye sourdough. Sourdough microbiota consist of fructan-degrading strains of the *L. delbrueckii* group and heterofermentative strains of the *L. reuteri* group, which convert fructose to mannitol. Drawn with data from [65].

Glucose and maltose levels in wheat and rye sourdoughs and consequently carbon catabolite repression in homofermentative lactobacilli and yeasts [38,41] are determined by the level of damaged starch and the β-amylase and amyloglucosidase activity in flour (Figure 1; [18,64]). If enzyme activity and the level of damaged starch in flour are low, sucrose, raffinose, and fructans become the most readily available carbohydrates [64]. The composition of the microbiota in rye sourdoughs that are low in damaged starch match the composition in other type II sourdoughs with organisms of the *L. delbrueckii* group including *L. crispatus*, *L. amylovorus*, and *L. ultuensis*, and organisms of the *L. reuteri* group including *L. frumentii* and *L. pontis* as the dominant members [32,35,65]. The restricted availability of maltose and glucose, however, selects for strains expressing an exceptional fructanase (Figure 2, [62,65]). The prevailing enzyme activity is an extracellular exofructanase (Figure 2), which exhibits more than 80% of the maximum activity in the pH range of 4–6 and the temperature range of 30–60 °C [62]. Fructan hydrolysis in sourdough releases fructose that is partially converted to mannitol by *L. reuteri* group organisms (Figure 3). However, the restriction of carbohydrate sources also allows for mannitol conversion after fructans are completely consumed (Figure 3) and results in a virtually zero FODMAP sourdough. The use of this zero FODMAP sourdough in low FODMAP rye bread making involves the addition of unfermented rye flour, which is fermented for only a short time [65]. Nevertheless, the choice of appropriate raw materials and the use of FruA-positive and mannitol-fermenting lactobacilli allows fructan degradation in rye and rye sourdoughs for the production of bread with a low content of fructans and mannitol but a comparable fiber content when compared to regular bread [66,67,68].

## 5. Proof of Concept from Clinical Trials with Low FODMAP Rye Bread

Two clinical trials done with IBS patients verified that low FODMAP rye bread made by using the above described zero FODMAP sourdough influences the gastrointestinal symptoms and the extent of gas production generated in intestinal fermentation. In the first study in a randomized double-blind controlled crossover study, it was shown that low FODMAP rye bread caused less flatulence, less abdominal pain, fewer cramps, and less stomach rumbling than regular rye bread [66]. Of note, the low FODMAP bread retained a high dietary fiber content (10 g/100 g) although the FODMAP levels were lowered to one third [66]. Including the low FODMAP rye bread thus also increased the dietary fiber intake to the recommended level in IBS patients, avoiding drawbacks of the other low FODMAP diets [15]. A second randomized double-blind controlled crossover study evaluated the amount of breath hydrogen levels after consuming low FODMAP rye bread or regular rye bread [68]. Low FODMAP rye bread reduced the generation of hydrogen by colonic fermentation [68]. This study showed that significant differences between bread types may occur in their postprandial effects.

## 6. Conclusions and Future Directions

Conventional sourdough baking reduces and converts FODMAPs in rye and wheat flour; however, the extent of FODMAP reduction is dependent on the fermentation organisms, the fermentation process, the grain raw material, and the sourdough dosage to the final bread dough. The production of low FODMAP bread requires extracellular fructanase activity; sourdough fermentation with lactobacilli expressing fructanases or the use of fructanase-positive yeasts provide wheat or rye breads with a low FODMAP content. Low FODMAP bread can help to restrict the intake of FODMAPs but at the same time increase the intake of slowly fermentable dietary fiber in IBS patients. High fiber/low FODMAP bread likely prevents the depletion of intestinal bifidobacteria that has been observed on other low FODMAP diets [14,15] and shows promise in reducing symptoms of IBS.

Anecdotal evidence links sourdough bread to improved tolerance of wheat in individuals with non-celiac wheat sensitivities [69]. In addition to the degradation of FODMAPs during sourdough fermentation, reduction and degradation of wheat amylase trypsin inhibitors may improve wheat tolerance in some individuals [67]. Amylase trypsin inhibitors are suggested to play a role in intestinal and extra-intestinal symptoms as they induce inflammatory reactions [70]. Amylase trypsin inhibitors are highly disulfide-bonded proteins; reduction of disulfide bonds reduces bioactivity and accelerates proteolytic digestion. Sourdough fermentation generates reducing conditions and supports reduction and hydrolysis of highly disulfide-bonded proteins that resist digestion in unfermented dough [71]. A pilot trial recruiting IBS patients with non-celiac wheat sensitivity, however, showed no improvement of intestinal symptoms after consuming sourdough wheat bread compared with industrial wheat bread [67]. Difficulties in identifying the protective effects of sourdough fermentation in non-celiac wheat intolerance relate to the poorly identified and likely multifactorial triggers of (self-diagnosed) non-celiac wheat sensitivity, and the inherent difficulties in blinding consumption of wheat or wheat sourdough products in clinical trials [67]. Despite the lack of support from clinical trials, sourdough-derived solutions likely play a significant role when developing healthier bakery products for people with non-gluten wheat sensitivities.

## Figures and Tables

**Table 1:** Content of oligosaccharides and non-starch polysaccharides (%) in wheat and rye grains.

Saccharide	Wheat	Rye
Arabinoxylans	6–7	7–12
β-Glucans including lignified cellulose	0.3–3	2–3
Pectin	trace	trace
Mannans, galactans, and galacturonans	1–1.5	n.d.
Fructans	1–2	4.3–5
1-Kestose	0.1	0.3
Nystose	0.03	0.1
Sucrose	0.6–1.0	1.2–1.8
Maltose	trace	trace
Raffinose	0.2–0.7	0.1–0.7
Stachyose	trace	trace

Compiled with information from [17,23,24,25,26,27,28,29,30,31]; n.d., not determined.
